# Identification of micro-RNA networks in end-stage heart failure because of dilated cardiomyopathy

**DOI:** 10.1111/jcmm.12096

**Published:** 2013-09-02

**Authors:** Xiaoming Zhu, Hongjiang Wang, Fan Liu, Li Chen, Weijia Luo, Pixiong Su, Weiming Li, Liping Yu, Xinchun Yang, Jun Cai

**Affiliations:** a Department of Cardiology Beijing Chaoyang Hospital, Capital Medical UniversityBeijing, China; b Center of Clinical Laboratory Zhongshan Hospital Medical College of Xiamen UniversityXiamen, China; c Stem Cell Engineering Laboratory Texas Heart InstituteHouston, TX, USA; d Center for Molecular Medicine and Experimental Therapeutics Department of Biology and Biochemistry, University of HoustonHouston, TX, USA

**Keywords:** heart failure, microRNAs, network

## Abstract

Micro-RNAs regulate gene expression by directly binding to the target mRNAs. The goal of the study was to examine the expression profiling of miRNAs in human failing hearts and identify the key miRNAs that regulate molecular signalling networks and thus contribute to this pathological process. The levels of miRNAs and expressed genes were analysed in myocardial biopsy samples from patients with end-stage heart failure (*n* = 14) and those from normal heart samples (*n* = 8). Four networks were built including the Gene regulatory network, Signal-Network, miRNA-GO-Network and miRNA-Gene-Network. According to the fold change in the network and probability values in the microarray cohort, RT-PCR was performed to measure the expression of five of the 72 differentially regulated miRNAs. miR-340 achieved statistically significant. miR-340 was identified for the first time in cardiac pathophysiological condition. We overexpressed miR-340 in cultured neonatal rat cardiomyocytes to identify whether miR-340 plays a determining role in the progression of heart failure. ANP, BNP and caspase-3 were significantly elevated in the miR-340 transfected cells compared with controls (*P* < 0.05). The cross-sectional area of overexpressing miR-340 cardiomyocytes (1952.22 ± 106.59) was greater (*P* < 0.0001) than controls (1059.99 ± 45.59) documented by Laser Confocal Microscopy. The changes of cellular structure and the volume were statistical significance. Our study provided a comprehensive miRNA expression profiling in the end-stage heart failure and identified miR-340 as a key miRNA contributing to the occurrence and progression of heart failure. Our discoveries provide novel therapeutic targets for patients with heart failure.

## Introduction

Heart failure, a main cause of morbidity and mortality worldwide, is the end stage of many cardiovascular diseases [1]. As the population ages, the cost associated with cardiovascular diseases are expected to increase substantially [2]. Although hundreds of genes relevant to various functions have been reported to play important roles in the development of heart failure [3]. The mechanisms are not fully understood mainly because of the complexity of the disease, especially the intricate regulation of gene expression. Bioinformatics analysis may contribute to solving this problem [4]. A major application of bioinformatics, since the late 1980s, has been in the areas of genomics and genetics, particularly in genomic studies involving large-scale DNA sequencing. Identification of a miRNA/gene interaction network may lead to a better understanding of the gene regulation that occurs during heart failure [5].

miRNAs are small non-coding RNAs of 21-25 nucleotides that usually negatively modulate gene expression at the post-transcriptional level *via* incomplete or complete complementary binding to target sequences within the 3′ untranslated region of mRNA [6]. MiRNAs have been identified in most types of cells and tissues. Approximately 10–30% of genes [7], especially those related to signal transduction [8–10], may be regulated by miRNAs. Moreover, multiple studies have suggested that miRNAs are involved in a variety of biological processes such as inflammatory responses [11], cell proliferation [12], development [13], differentiation [14], apoptosis [15] and tumorigenesis [16]. Aberrant and absent expression of miRNAs were often associated with pathophysiology disorders [17, 18]. Chen *et al*. [19] reported that cardiac-specific knockout of Dicer, a gene encoding an RNase III endonuclease essential for miRNA processing, leads to rapidly progressive dilated cardiomyopathy (DCM), heart failure and post-natal lethality. Therefore, we suggested that miRNAs play a significant role in regulating globally molecular signalling networks during remodelling and the transition to heart failure. Combined miRNA and mRNA profiling may potentially provide diagnostic and prognostic information in the end-stage cardiomyopathy [20]. These findings suggest the important role of miRNAs in the global regulation of cardiac function.

The aim of our study was to compare the expression profile of miRNAs and mRNAs in myocardial biopsy from end-stage DCM patients with that in normal myocardial samples. Taken into consideration, the global regulatory role of miRNAs, many target genes may be regulated concomitantly by a single miRNA whereas a single gene could be the target of multiple miRNAs [21]. It was difficult to focus on the effects of specific miRNAs and individual genes because of the complexity of the regulatory process. miRNA microarrays have become an important technology platform because they are high throughput, miniaturized and capable of parallel analysis. [22] Thus, miRNA microarrays were used in this study for the global analysis of biological systems. Our goal was to delineate global changes in miRNAs and gene expression between failing and non-failing human hearts to define key molecules and discover molecular pathways as novel mechanisms involved in the progression of heart failure. The miRNA microarray data were verified by quantitative realtime-PCR (qRT-PCR). Bioinformatics analysis was used to demonstrate functional pathways. Our study indicated an intrinsic correlation between miRNAs and their target genes, and provided new insight into the mechanisms involved in the progression of heart failure.

## Materials and methods

### Patients and tissue samples

The study protocol was approved by the Medical Ethical Committee of the Chaoyang Hospital of Capital Medical University, Beijing, China, and informed consent was obtained from each patient. Tissue for analysis was collected from the LV free wall of explanted hearts from patients undergoing heart transplantation with a diagnosis of DCM (EF ≤ 35%; *n* = 14), and from unmatched healthy donors whose hearts were not suitable for transplantation (*n* = 8). The transplant recipients and healthy donors were all Han people. The hearts were arrested and transported to the laboratory in an ice-cold, oxygenated cardioplegic solution. In the laboratory, the tissue was flash frozen in liquid N_2_ and stored at −80°C.

### RNA isolation

Total RNA was harvested using TRI Reagent BD (Sigma-Aldrich, St. Louis, MO, USA) and an RNeasy Mini Kit (Qiagen, Valencia, CA, USA) according to the manufacturer's instructions. In detail, 100 mg of LV tissue was homogenized in TRI Reagent BD, and incubated at room temperature for 5 min. Chloroform was added to the samples, and mixed vigorously followed by incubation at room temperature for 5 min. The samples were then centrifuged at 12,000 × g for 15 min. at 4°C. The aqueous phase containing the RNA was removed carefully, and RNA was precipitated with 100% ethanol. The mixture was applied to an RNeasy Mini spin column and washed several times, and RNA was eluted by adding 25 μl RNase-Free Water. RNA was stored at −80°C until further processing.

### Gene expression array

RNA samples were labelled using the Illumina labelling kit and hybridized on the Illumina Human WG-6-v3 Expression BeadChip Array. The HumanWG-6 v3.0 Expression BeadChip contains six arrays on a single BeadChip, each with >48,000 probes derived from human genes in the NCBI RefSeq and UniGene databases. HumanWG-6 Expression BeadChips used the Direct Hybridization Assay and were scanned on an Illumina iScan. GenePix pro V6.0 software was used to read the raw intensity of the image [23]. Perfectly matched fluorescence intensities were background-corrected, mismatch-adjusted, normalized and summarized. The GC Robust Multi-array Average (GCRMA) algorithm was used to calculate log2-transformed gene expression data [24]. The threshold value of define up- and down-regulated mRNAs was a fold change greater than 1.5, and Student's *t*-test *P* < 0.05 was considered significant.

### miRNA array

**Fig. 1:**
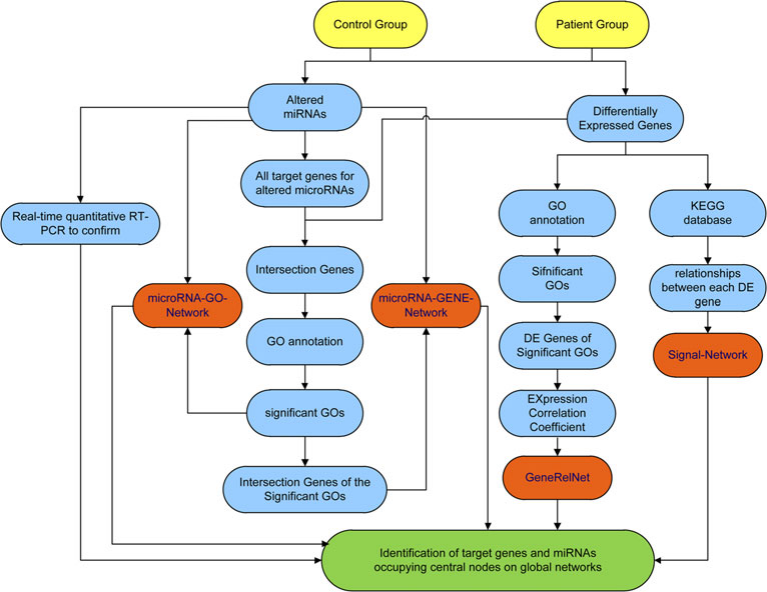
Schematic depiction of the experimental design and flowchart of the steps performed in this study.

The experimental procedure of differential expression of miRNAs in control group and patient group was described in Figure 1. The Illumina MiRNA Expression Profiling Assay system was used to perform miRNA expression profiling of tissue samples from 14 patients with DCM and eight healthy donors. RNA samples were labelled following the Illumina TruSeq Small RNA Sample Preparation protocol and hybridized in the Illumina miRNA Expression Profiling Assay system. Total RNA was mixed with biotinylated oligonucleotide primers. After incubation, the first strand was synthesized using Superscript II RNaseH-reverse transcriptase. The cDNA was then incubated at 65°C to denature the RNA/DNA hybrids and degrade the RNA templates. The labelled targets then were subjected to chip hybridization. Hybridization was carried out on the miRNA microarray with the Illumina miRNA Expression Profiling Assay system containing 735 assays for detecting 470 miRNAs, as described in the miRBase database v9.1, and 265 potential miRNAs, as identified in a RAKE analysis study [25]. Often, more than one probe set was used for one mature miRNA, and quadruplicate probes corresponded to most precursor miRNAs. Detection of biotin-containing transcripts was performed with streptavidin–Alexa Fluor 647 conjugates and by examination of scanned images (Axon 4000B), which were quantified using GenePix 6.0 software (Axon Instruments, Inc., Union City, CA, USA). The intensity of the green signal was calculated after background subtraction, and replicated spots on the same slide were averaged to obtain the median intensity. The median normalization method was used to normalize the data (which equals Foreground/Background median). The median was the 50th percentile of the miRNA intensity and was greater than 50 in all samples after background correction. The threshold value of up-regulated or down-regulated miRNAs was a fold change greater than 1.5, and Student's *t-*test *P* < 0.05 was considered significant. The miRNAs selected for investigation in our study, which were called altered miRNAs, were further filtered based on their expression levels.

After identification of altered miRNAs from the microarray data, the predicted targets for the altered miRNAs were identified using TargetScan 5.1 database. The intersection of identified genes was obtained based on the data in the databases and the differentially expressed (DE) genes.

### Bioinformatics of heart failure

#### DE genes

A specific cut-off value of 1.5 times greater was set to identify genes, the expression of which was significantly and differentially regulated. These genes were called DE genes. Differences in DE genes between the control and DCM samples were identified by Student's *t*-test. Statistical significance was set as *P* value cut-off lower than 0.05.

#### The gene ontology database and the kyoto encyclopaedia of genes and genomes database

Significant progress in data mining has provided a wide range of bioinformatics analysis tools, including the gene ontology (GO) and kyoto encyclopaedia of genes and genomes (KEGG) databases. The GO database is a relational database comprising the gene ontologies, the annotations of genes and gene products to terms. The advantage of combining the ontologies and annotations in a single database is that powerful queries can be performed over annotations using the ontology.

The KEGG database (http://www.genome.ad.jp/kegg/) provides searchable pathways for molecular interactions and reaction networks for metabolism, various cellular processes and multiple human diseases. The relationships between each DE gene and the others were obtained from the KEGG database.

### Networks

#### Gene regulatory network (GeneRelNet) and signal-network

Annotations of DE genes between control and DCM samples were made based on the GO database. These genes were classified into hierarchy, and gene products with similar functions were placed together. All GOs were tested with Fisher's exact test and Chi-Square test to determine the significance level and false discovery rate (FDR). Then, genes were identified as significant GOs with *P* < 0.01 and FDR <0.05. The expression correlation coefficients between genes with the significant GOs were calculated separately for the failing and non-failing samples. The correlation of the genes in the network was measured as the degree. A higher degree indicated more correlation with other genes. The constellation graphical clustering method [26] for creating the gene co-expression network was based on the correlation of the gene expression pattern in the control and heart failure groups.

Signal relationships between the DE genes were based on the KEGG database. The constellation graphical clustering method for creating the signalling network was based on the correlation of each DE gene with the other genes.

#### miRNA-GO-network and miRNA-GENE-network

We obtained the intersection between the predicated target genes of altered miRNAs and the DE genes we identified. The intersection genes were annotated based on the GO database. All annotations were tested with Fisher's exact test and Chi-Square test to obtain the significance and FDR. We then obtain both the significant GOs and the genes belonging to the significant GOs with *P* < 0.01 and FDR <0.05.

The miRNA-Gene-Network was based on the targeting regulatory relationships between the miRNAs and the intersection genes, using graph theory with Cytoscape Version 2.6.0 software (http://www.cytoscape.org/). The miRNA-GO-Network was constructed with both the significant GOs from the intersection genes and altered miRNAs.

#### Reference in the network graphs

These networks were the graphical representations of the relationships between the gene, GO and miRNA. Genes, gene products, GO or miRNAs were represented as nodes in the graph, and the biological relationship between two nodes is represented as a line. All lines were supported by at least one reference from the published literature, textbook or the functional information in the GO or KEGG database. Nodes were displayed using various shapes that represent the functional class of the gene product. Lines were displayed with labels that describe the relationship between the nodes. We could then infer key roles of a relationship or regulation in the networks. The size of the nodes indicated the degree of the relationship in the network. A larger node indicated more connections with other genes, and thus, this node might play a more important role in the network.

### Real-time quantitative reverse transcription (RT)-PCR

First strand cDNA was synthesis from RNA by using MMLV reverse transcriptase (Epicentre, Madison, WI, USA) according to the manufacturer's instructions. SYBR-green based quantitative PCR (qPCR) was performed on an ABI PRISM 7500 system (Applied Biosystems, Foster City, CA, USA). The relative expression level of each miRNA was normalized to the internal control small nuclear U6 expression and calculated by the ΔΔCt method. Selected miRNAs (hsa-miR-10a, miR-19b, miR-181c, miR-302d and miR-340) were further quantified with TaqMan qRT-PCR. Each reaction was primed using a gene-specific stem-loop primer. Selected RNAs (ANP, BNP, caspase-3) were quantified by SYBR-green-based qRT-PCR. The RT stem-loop primers, the PCR primers and the probes are listed in Tables 1 and 2.

**Table 1:** Sequences of the primers and probes used in the TaqMan RT-PCR validation (From Applied Biosystems)

Assay ID	Assay name	Mature microRNA sequence
387	hsa-miR-10a	UACCCUGUAGAUCCGAAUUUGUG
2258	hsa-miR-340	UUAUAAAGCAAUGAGACUGAUU
482	hsa-miR-181c	AACAUUCAACCUGUCGGUGAGU
396	hsa-miR-19b	UGUGCAAAUCCAUGCAAAACUGA
535	hsa-miR-302d	UAAGUGCUUCCAUGUUUGAGUGU

F: female; M: male; LVEF: left ventricular ejection fraction measured prior to explant; DCM: dilated cardiomyopathy (pre-transplant diagnosis). Drug therapy: DIG: digoxin; DOB: dobutamine; AMIO: amiodarone; ACEI: angiotensin converting enzyme inhibitor (usually lisinopril); BB: β-adrenergic blocker (metoprolol or carvedilol).

**Table 2:** Sequences of the primers used in the SYBR-green-based quantitative RT-PCR validation

Primer name	Primer sequence
ANP: Forward	5′GGGCTTCTTCCTCTTCCTGG3′
ANP: Reverse	5′GCAGATCTATCGGAGGGGTC3′
BNP: Forward	5′TCCAGAACAATCCACGATGC3′
BNP: Reverse	5′AAACAACCTCAGCCCGTCAC3′
caspase-3: Forward	5′GAACGAACGGACCTGTGGA3′
caspase-3: Reverse	5′CGGGTGCGGTAGAGTAAGC3′

The PCR reactions were carried out in a final volume of 20 μl using a 7500 real time-PCR System (Applied Biosystems). Reactions consisted of 1.33 μl cDNA, TaqMan Universal PCR Master mix (Applied Biosystems), 0.2 μM TaqMan Probe, 1.5 μM forward primer and 0.7 μM reverse primer. The PCR reactions were initiated with 10 min. incubation at 95°C followed by 40 cycles of 95°C for 15 sec. and 60°C for 60 sec. The absolute expression levels of miRNAs were normalized to the internal control small nuclear U6 and were calculated by the ΔΔCt method.

### Cell culture and transfection with lentivirus

Neonatal rat ventricular myocytes were isolated from 1-day-old Sprague Dawley rats as described [27]. All experimental procedures were approved by the Institutional Animal Care and Use Committee of Capital Medical University. Neonatal hearts were placed in an ice-cold Hanks balanced salt solution (HBSS). The apex of the ventricular from the lower 1/3 of the heart was separated and cut into smaller pieces. The pieces were incubated in 5 ml of digestion buffer (1 mg/ml trypsin and 0.75 mg/ml collagenase in HBSS), and gently stirred at room temperature for 10 min. The digest was collected in 5 ml high glucose DMEM with 20% heat-inactivated Foetal Bovine Serum (FBS). The digestion process was repeated six times. The cells were then centrifuged at 283 × g for 5 min. and re-suspended in serum-containing media (10% FBS, 1% penicillin-streptomycin, high glucose DMEM). The cells were pre-plated in 100 mm TC-Treated Culture Dish (Corning® #430167) for 1 hr to attach non-cardiac cells. The density of non-attached cells was counted using a haemocytometer and plated into 6-well culture plates at a density of 1 × 10^6^ cells per well. After 48 hrs, the culture medium was replaced with high glucose DMEM containing 2% FBS and 5-Bromo-2′-deoxyuridine (BrdU; 100 mM).

**Fig. 2:**
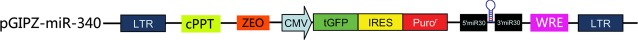
Lentiviral (pGIPZ) constructs used to deliver miR-340 expression in cardiomyocytes.

To generate a miR-340 expression vector, a ≍260-bp genomic fragment up and downstream of the pre-mir-182 form was amplified by PCR and the fragment containing strictly the pre-miR-340 form was cloned into pGIPZ (Open Biosystems, Pittsburgh, PA, USA) that allows regulated expression of miR-340 upon zeocin treatment. The vector was sequenced to confirm insert direction and sequence identity (Fig. 2). Then, e-GFP alone or e-GFP followed by miR-340 (SunBio Medical Biotechnology Company, Shanghai, China) was added to the medium and the cultures were incubated for the indicated time. Forty-eight hours later, the green fluorescence of e-GFP could be visualized with the fluorescence microscope. Ninety-six hours after transfection, RNA was extracted to evaluate ANP, BNP and caspase-3 expression.

### Laser confocal scan of myocardial cells transfected with lentivirus

For the cross-sectional areas scan, the coverslips were put into each well of 6-well culture plate before seeding the cells. The cells on the coverslips were washed three times with PBS, fixed with 3.7% formaldehyde in PBS, permeabilized in 0.1% Triton X-100 in PBS. Cells were then stained with α-actinin (Sigma-Aldrich, 1:100 at 4°C) overnight. The coverslips were incubated with red fluorescence conjugated secondary antibody (1:1000; Santa Cruz Biotechnology, Inc., Dallas, TX, USA) for 1 hr at room temperature. The nucleus was stained with DAPI for 1 min. Cell cross-sectional area (pixel 0.763 μm) was documented by Laser Confocal Microscopy and compared between miR-340-transfected myocardial cells and control cells.

### Statistical analysis

In this study, the increase or decrease of more than 1.5-fold of the expressed gene or miRNA was the designated standard for distinguishing changes and analysis. Data were expressed as the mean ± SD. All statistical analyses were performed with SPSS version 13.0. Functional data were analysed using repeated measures anova (with a *post hoc* Tukey test for pairwise comparisons), Fisher's exact test or Chi-Square test. Statistical significance was set at a *P* < 0.05.

## Results

### Patient population and heart samples

Clinical parameters for all patients are described in Table 3. To obtain a global expression pattern of the genes and miRNAs involved in end-stage heart failure, total RNA was isolated from the hearts of 14 patients (*i.e*. explanted hearts of transplant recipients with a diagnosis of DCM) and eight healthy hearts. The LV ejection fractions (LVEFs) in all failing hearts were ≤35%, and in all non-failing hearts were >55%. The mean age of the patients with DCM was 40.5 ± 8.7 years and of donors with non-failing hearts was 41.0 ± 8.2 years. All patients with heart failure met the World Health Organization diagnostic criteria for DCM.

**Table 3:** Clinical characteristics of patients with heart failure

ID no.	Gender	Age (years)	Disease	LVEF (%)	Medications
1	M	33	DCM	14	DIG, ACEI
2	F	45	DCM	35	ACEI, BB
3	M	43	DCM	34	AMIO, ACEI
4	M	39	DCM	35	BB, ACEI
5	M	57	DCM	33.9	ACEI, AMIO
6	M	38	DCM	31.7	DIG, ACEI
7	M	48	DCM	30.4	ACEI, BB
8	M	42	DCM	26.3	DIG, DOB
9	F	50	DCM	35	ACEI, AMIO
10	M	26	DCM	31	DIG, ACEI
11	M	29	DCM	25	AMIO, ACEI
12	M	38	DCM	22	BB, ACEI
13	F	32	DCM	31	ACEI, AMIO
14	M	47	DCM	34	DIG, ACEI

### GeneRelNet and signal-networks

**Fig. 3:**
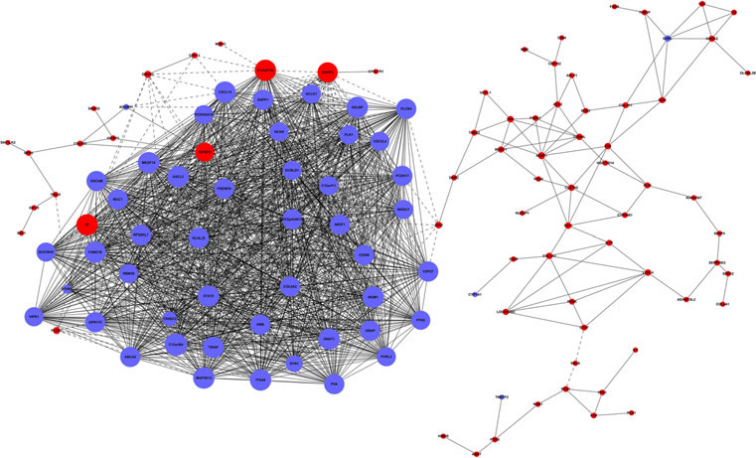
Gene regulatory networks of the normal group. The red round spots are up-regulated genes, and the blue round spots are down-regulated genes. The lines represent positive correlation between genes. Dotted lines represent negative correlation between genes. Genes regulated by more other genes are represented by larger spots.

**Fig. 4:**
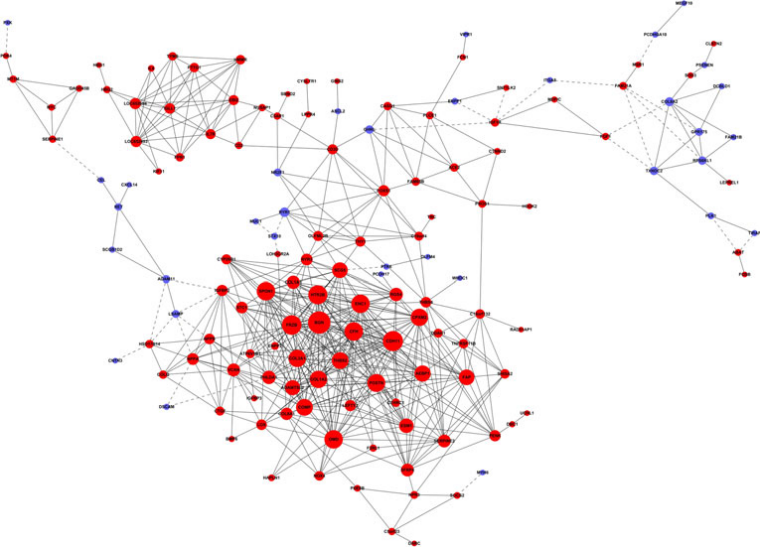
Gene regulatory networks of the heart failure group. The red round spots are up-regulated genes, and the blue round spots are down-regulated genes. The lines represent positive correlation between genes. Dotted lines represent negative correlation between genes. Genes regulated by more other genes are represented by larger spots.

A total of 733 DE genes were identified by comparing gene expression profiles between control and DCM samples. Gene ontology analysis of the DE genes showed that 131 genes were up-regulated and 35 genes were down-regulated in DCM samples. The known functions of up-regulated genes included liver immune response, positive regulation of the acute inflammatory response, asymmetric protein localization, γ-aminobutyric acid metabolism and others, whereas those of the down-regulated included the negative control of cyclic nucleotide phosphodiesterase, vitamin transport, negative regulation of neurogenesis and others. To evaluate the relationship between the genes and significant GOs, expression correlation coefficients were calculated separately for the failing and control samples. The GeneRelNet for each group (Figs 3 and 4) was constructed based on the expression correlation coefficients. The correlation levels of genes in the network were indicated in Table 4. The importance of the gene in the GeneRelNet was indicated by the clustering coefficiency, which referred to the density of one gene with adjacent genes. The genes with the highest degrees were C12orf64 with 51 degrees, followed by FAM21B, ITGA8, MAP3K14, MEGF10, STX10, TIRAP, ASCL2, GNAT1, INSM1, HDLBP, CXCL14, TF and C14orf132 each with 50 degrees. In the DCM group, the genes with the highest degrees were AEBP1, FAP and POSTN, with 16 degrees.

**Table 4:** Difference of degrees between the DCM GenRelNet and Control GenRelNet

Gene	Gene degree of the disease network	Gene degree of the control network	Difference of degrees
ITGA8	1	50	-49
STX10	1	50	-49
TIRAP	2	50	-48
CEP27	2	49	-47
ENPP1	2	49	-47
MEGF10	3	50	-47
NR2F1	2	49	-47
WHDC1	1	48	-47
C14orf132	4	50	-46
DKFZp434K191	3	49	-46
GBAP	3	49	-46
NCAN	2	48	-46
PLK1	3	48	-45
RYR1	1	46	-45
ASCL2	6	50	-44
COL8A2	5	49	-44
GPR175	5	49	-44
IGFBP2	6	48	-42
RPS6KL1	7	49	-42
DCBLD1	9	48	-39
C12orf11	10	48	-38
TXNDC2	9	44	-35

**Fig. 5:**
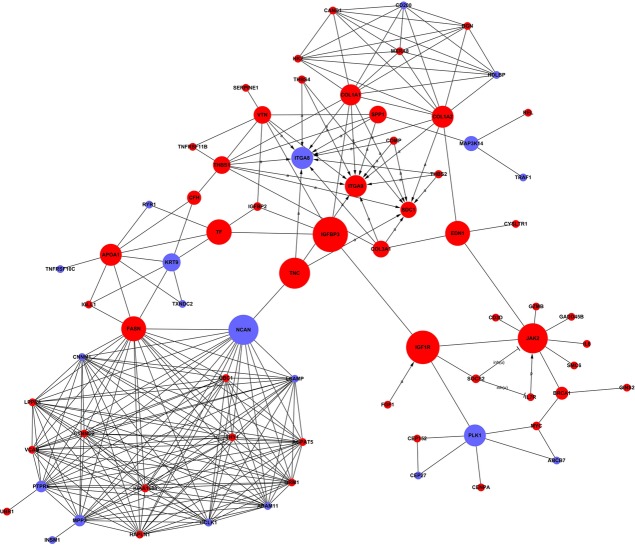
Signal-Network. Round spots represent the genes. The lines show the interactions between genes. The arrows represent activation, and the flat head on behalf of inhibition. The symbols on the lines show the action between genes. a: activation, inh(u): inhibition.

To evaluate the potential underlying molecular signalling pathways involved, a signal-network (Fig. 5) was constructed using the relationships in the KEGG database. The ‘betweenness centrality’ was used to measure the intermediary capacity of each gene [28]. Some important genes in the pathway may affect others. The key genes were identified in the signal-network according to the ranking of the degree and betweenness centrality. The genes with the top degrees in the network were FASN (19 degrees) and NCAN (17 degrees). However, IGFBP3 (betweenness centrality = 0.259) and IGF1R (betweenness centrality = 0.248) showed the most critical intermediary capacity in the network.

### miRNA-GO-network and miRNA-gene-network

#### Specific expression profiles of miRNAs in end-stage DCM samples

miRNA expression profiles in myocardial samples from 14 DCM patients and eight healthy donors were evaluated by the Illumina miRNA Expression Profiling Assay system. RNA from each patient was considered as independent sample; therefore, the miRNA microarray data set obtained showed independent expression of each individual. The levels of 98 miRNAs were compared between patients and healthy controls. The unsupervised clustering of the expression profiles of cardiac miRNAs in cardiomyopathy and non-failing hearts revealed a distinct miRNA profile associated with DCM.

#### Predicted targets of altered miRNAs

**Fig. 6:**
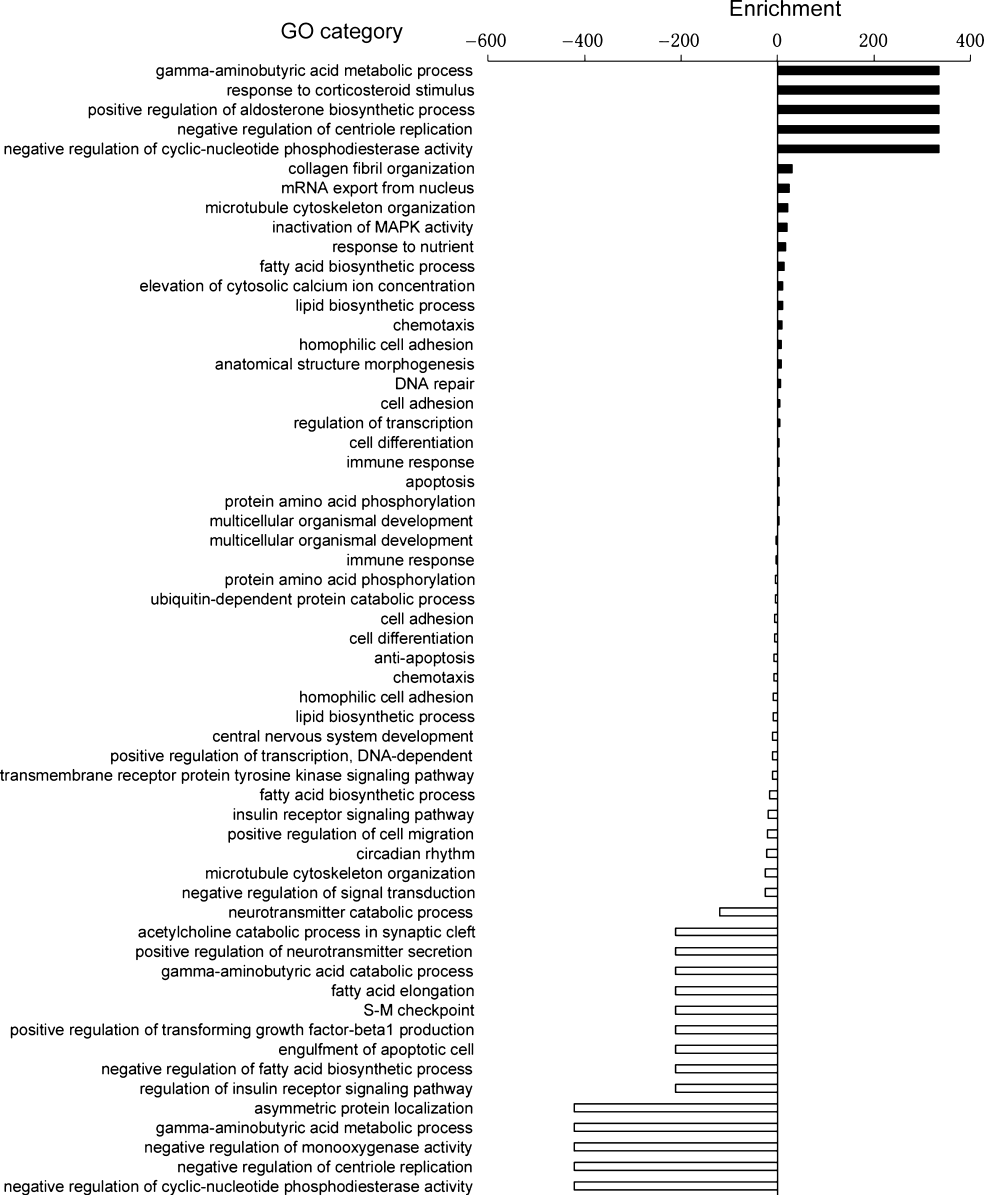
Significant GO of altered miRNAs. The black bars indicate greater enrichment of GOs, and the white bars indicate lesser enrichment of GOs.

As alteration of these miRNAs occurs simultaneously and influences their respective targets, the global effect is a sum of the effects coordinated by individual miRNAs. Of the 735 miRNAs detected in the microarray, 57 were up-regulated whereas 41 down-regulated in DCM samples when compared with samples from healthy donors (*P* < 0.05). Using the TargetScan 5.1 database, a total of 22,074 genes were predicted targets for the altered miRNAs in DCM. Among them, 153 out of the 733 DE genes were predicted as targets of 72 altered miRNAs in DCM. Therefore, the networks were built based on these 72 miRNAs. The intersection genes were annotated in the GO database. We found 58 significant GOs (*P* < 0.01; Fig. 6), with 24 up-regulated and 34 down-regulated.

### Network construction

**Fig. 7:**
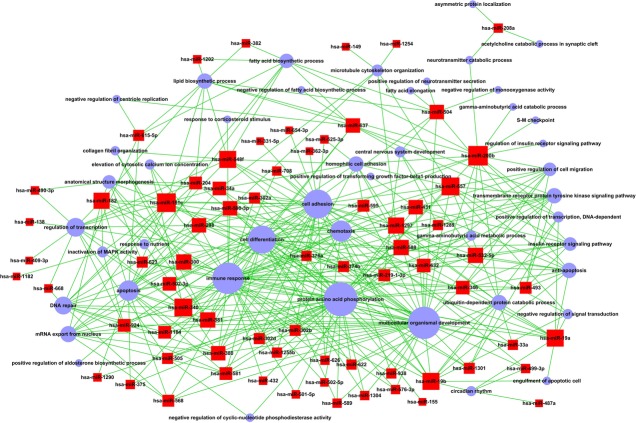
MiRNA-GO-Network. The red squares are miRNAs, and the blue round spots are GOs. The lines represent the interactions between miRNAs and GOs. GOs regulated by more miRNAs are represented by larger spots. Similarly, miRNAs that regulate more GOs are represented by larger squares.

To better understand the connections between miRNAs and GOs, a miRNA-GO-Network (Fig. 7) was built with the significant GOs and the altered miRNAs using Cytoscape Software. The degree of any particular miRNA was determined by the number of GOs regulated by the miRNA in the network. The miRNAs hsa-miR-200b (16 degrees), hsa-miR-181c (14 degrees), hsa-miR-340 (13 degrees), hsa-miR-557 (13 degrees), hsa-miR-19a (12 degrees), hsa-miR-19b (12 degrees) and hsa-miR-548f (12 degrees) were significantly differentially regulated in DCM samples compared with non-failing control samples. The important GOs, defined as the most highly regulated by the altered miRNAs, were involved in protein phosphorylation (42 degrees), multicellular organismal development (40 degrees) and immune responses (39 degrees).

**Fig. 8:**
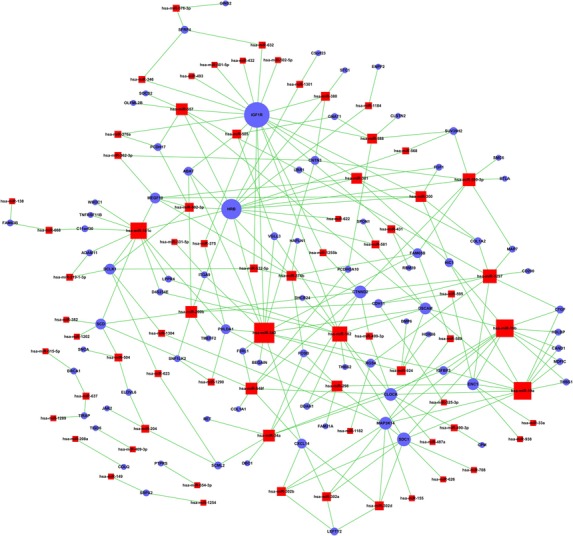
miRNA-Gene-Network. The red squares are miRNAs, and the blue round spots are genes. The lines represent the interactions between miRNAs and genes. Genes regulated by more miRNAs are represented by larger spots. Similarly, miRNAs that regulate more genes are represented by larger squares.

The miRNA-Gene-Network (Fig. 8) built with Cytoscape was based on the target regulatory relationships between the miRNAs and the intersection genes. The miRNAs hsa-miR-19a (12 degrees) and hsa-miR-19b (12 degrees) were significantly down-regulated in the DCM samples. In contrast, the miRNAs hsa-miR-340 (14 degrees), hsa-miR-181c (11 degrees) and hsa-miR-182 (10 degrees) were significantly up-regulated in the DCM samples. The miRNA degree represented the number of predicted targets regulated by any particular miRNA in the network. IGF1R (19 degrees) and HRB (14 degrees) were the most highly regulated genes.

### Real-time quantitative RT-PCR

**Fig. 9:**
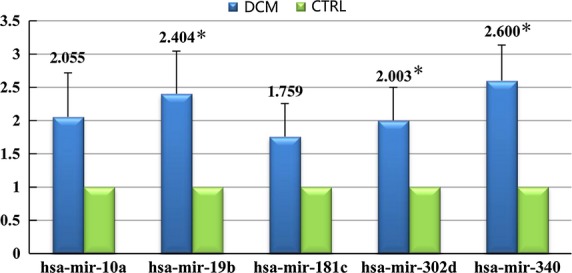
Quantitative RT-PCR for significantly altered miRNAs in DCM and control samples. Blue bars represent the DCM group, and green bars represent the control group (*P < 0.05).

To confirm the findings of the miRNA profiles and networks, qRT-PCR was performed to measure the expression of five of the 72 differentially regulated miRNAs, based on their fold change or degree in the network, probability values in the microarray cohort and importance in the network. The relative expression of miRNAs in DCM samples and healthy control samples is shown in Figure 9. We found a 2.6-fold increase in hsa-miR-340 expression (*P* < 0.001), a 2.4-fold increase in hsa-mir-19b expression (*P* < 0.01) and a twofold increase in hsa-miR-302 expression (*P* < 0.05) in DCM samples. In addition, hsa-miR-181c (*P* > 0.05) and hsa-miR-10a (*P* > 0.05) exhibited a trend of higher expression levels in DCM samples, but these differences did not reach statistical significance when compared with control samples (Fig. 9).

Van Almen *et al*. [29] reported that decreased hsa-miR-19 expression leads to increased expression of CTGF and TSP-1 in aged failure-prone hearts. The hsa-miR-19b and hsa-miR-302 were down-regulated in the profile analysis, but up-regulated in the quantitative RT-PCR assay. Then, we focused on the unique hsa-miR-340 in the miRNA-Gene-Network because it had the highest degree in DCM samples compared with normal control samples in both the profiling and the qRT-PCR assay (Fig. 9).

### Cell culture and transfection with lentivirus

**Fig. 10:**
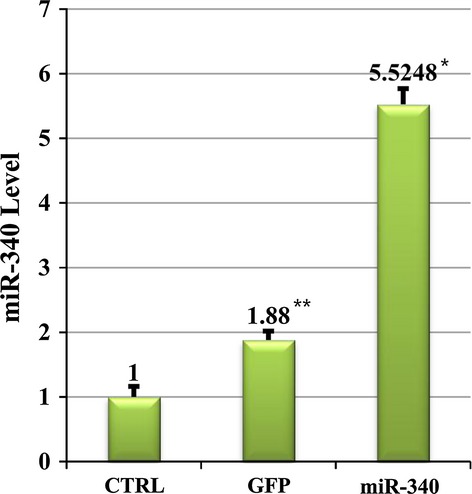
Quantitative RT-PCR for miR-340. There is a 5.52-fold increase in miR-340 expression in the cultured cardiomyocytes transfected with lentivirus than that in the controls (*P < 0.05; **P > 0.05 *versus* control).

**Fig. 11:**
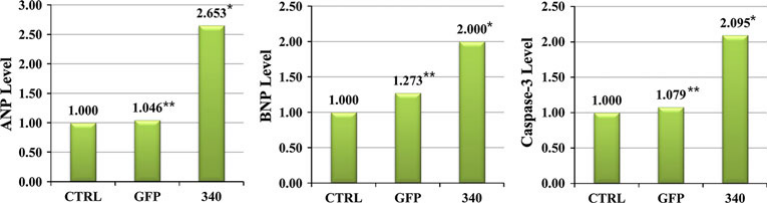
Quantitative RT-PCR for ANP, BNP and caspase-3. The levels of ANP, BNP and caspase-3 are significantly elevated in the miR-340 transfected cells compared with controls, while that are similar between the eGFP group and the control group (*P < 0.05; **P > 0.05 *versus* control).

It is important to determine if altered miRNA expression is the ‘cause’ or the ‘effect’ of heart failure. It is not feasible to identify alterations in miRNA profiles at the time of initiation of cardiac dysfunction in humans; therefore, cardiomyocytes isolated from neonatal rats were cultured to verify the alterations observed in the miRNAs we identified. The cardiomyocytes were transfected with lentiviral vector–encoded miR-340. The expression levels of miR-340 and the genes ANP, BNP and caspase-3, which are associated with the severity of heart failure and cardiomyocyte apoptosis, were examined by RT-PCR. We found a 5.52-fold increase in miR-340 expression (*P* < 0.05) in the cultured cardiomyocytes transfected with lentivirus (Fig. 10). The relative expression of the genes ANP, BNP and caspase-3 were, respectively, 2.653, 2.000 and 2.095 times greater in miR-340 transfected cells than that control (Fig. 11). The changes in these gene expressions were statistically significant.

### Laser confocal scan of myocardial cells transfected with lentivirus

**Fig. 12:**
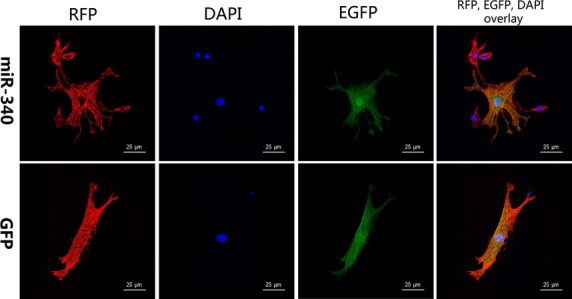
Laser confocal microscopy of myocardial cells transfected with e-GFP vector or Lentivirus encoding miR-340. The bottom row shows myocardial cells transfected with Lentivirus (only e-GFP, no miR-340), and the upper row shows myocardial cells transfected with Lentivirus (miR-340 overexpression). The nuclei are shown in blue, the Troponin in red and Lentivirus transfected cells in green.

We overexpressed miR-340 in cultured cardiomyocytes to validate its targets. Laser confocal microscopy showed that the cell structure and volume changed significantly in miR-340-transfected cardiomyocytes compared with those transfected with green fluorescent protein (GFP; Fig. 12). The cross-sectional area of cardiomyocytes was measured, including those transfected with e-GFP (*n* = 103) and miR-340 (*n* = 103). The cross-sectional area of miR-340-expressing cells (1952.22 ± 106.59) was significantly (*P* < 0.0001) greater than that of cells expressing GFP (1059.99 ± 45.59). Taken together, these data suggested that miR-340 plays a role in regulating nodal molecules in human DCM and that dysregulation of miRNAs may contribute to cardiac dysfunction.

## Discussion

MiRNAs regulate gene expression mostly through repression and providing system-wide influences on gene expression systems. MiRNA-mediated myocardium gene expression is one of the novel mechanisms proposed for heart failure. Recent studies have suggested the involvement of miRNAs in the regulation of hypertrophy and heart failure [30–32]. DCM is the net result of cross-talk between expressed genes and non-traditional gene regulation by miRNAs. Four networks were built from the predicted targets of these altered miRNAs. Thus, combinatorial expression patterns of miRNAs in DCM provide the theoretical framework for discerning the cooperative action of miRNAs on gene expression in human heart failure. Recent studies [33, 34] demonstrated that cardiac-specific deletion of dicer leads to significant reduction in mature miRNA levels, resulting in DCM and heart failure, which supports that miRNAs may play a critical role in globally regulating cardiac signalling and function.

High-throughput microarray analysis provides a global view of the miRNAs and molecules in cardiac disease. Thus, we examined the potential regulation of specific signalling networks by altered miRNAs in heart failure and built the GeneRelNet, signal-network, miRNA-GO-Network and miRNA-Gene-Network using Cytoscape Software. Genes that embodied significant GOs were further subjected to function diversity analysis of targeting to reflect differences between the heart failure group and the normal control group. Up-regulated GOs included liver immune response, positive regulation of acute inflammatory response and asymmetric protein localization of γ-aminobutyric acid metabolism. Down-regulated GOs mainly included negative regulation of cyclic nucleotide phosphodiesterase, vitamin transport, neurogenesis negative regulation and so on. Database search was used to identify differences in the interactions of genes with other genes between the two groups and the FASN and NCAN genes interacted most frequently with 19 and 17 other genes, respectively. IGFBP3 and IGF1R genes had intermediate capacity and were located in the key node in the Signal-Network. Analysis of the gene signal transduction pathway regulatory network (Signal-Network) demonstrated that network architecture within the normal group was more compact than the disease group, suggesting that the relationship between the heart failure related genes was damaged compared to the normal group, mainly because of down-regulation of gene expression. Thus, the networks become less compact and the amount of gene expression reduces in heart failure samples compared with normal samples.

These networks suggested that miRNAs are the major class of molecules associated with the progression of heart failure. The predicted targets regulated by the altered miRNAs mapped to the network of cardiovascular development and function. Bioinformatics analysis confirmed that these unrelated predicted targets of altered miRNAs are critical signalling components in the cardiovascular system, and provided an independent corroboration of our findings. By the gene regulatory network, differences in gene regulation between the two networks were determined and key genes were identified. Using gene mapping, we identified the key genes in the centre of the signal transduction network and some of the significant gene changes between the two groups disappeared. The remaining genes, with well represented sample differences, were used as the targets of our study.

One hundred and fifty-three DE genes were predicted as targets of the altered miRNA. In the network, the performance differences in the most critical miRNAs (hsa-miR-340, hsa-miR-19a, hsa-miR-19b, etc.) were because of the target genes IGF1R and HRB, which were frequently regulated by the miRNAs. IGF1R is widely involved in immune response, amino acid phosphorylation and anti-apoptotic biological pathways; whereas, HRB is involved in many biological processes, such as mRNA nuclear transport, the development of multicellular organisms, apoptosis, DNA repair and cell differentiation. Hsa-miR-340 regulated 26 target genes including IGF1R and HRB. At the same time, up-regulation of hsa-miR-181c and hsa-miR-182 targets HRB and IGF1R expression, suggesting the biological functions of the two key genes may be suppressed.

Analysis of miRNAs functions that were significantly differentially regulated and the intersection of genes in miRNA regulatory network identified miRNAs that play a central role in the network. The gene of amino acid protein phosphorylation, the development of multicellular organisms, the immune response and other functions are important for survival and development of the cardiomyocyte. The key miRNAs identified included hsa-miR-181c, hsa-miR-19a and hsa-miR-19b, which all have higher degrees in the network diagram. Real-time PCR demonstrated that hsa-miR-19b, hsa-miR-302d and hsa-miR-340 were significantly increased (*P* < 0.05), which validate the results from the miRNA array and implied an important role of miR-340.

These studies suggest that miRNAs may regulate unrelated signalling transduction networks. Therefore, the regulation of many signalling networks by miRNAs, rather than individual targets, shows the global scale of regulation. And miRNAs likely play a prominent role in altering the global signalling networks and pathways involved in the progression of cardiac hypertrophy and failure. The most important finding from our studies is the identification of a unique miRNA in human DCM that allowed us to define the global molecular signalling network regulated by miRNAs in cardiac pathology. The microarray platform, as well as the use of a relatively large set of human samples, led to the identification of miRNAs altered in DCM hearts compared with control human hearts. Importantly, our study identified miRNAs, hsa-miR-19b, hsa-miR-302d and hsa-miR-340 (Fig. 9) which were significantly up-regulated in human DCM and may play a critical role in the pathophysiology of heart failure. In addition, has-miR-181c and has-miR-10a showed a trend towards higher expression levels in DCM samples (*P* > 0.05). Previous studies show that miRNA-19a and 19b are members of the miR-17-92 cluster, which regulates the expression of ECM proteins CTGF and TSP-1 in the ageing-related heart failure process [35]. The miRNA-302 family plays a role in regulating growth factor signalling pathways, with implications for nephropathic cell fate transitions [37]. Androulidaki *et al*. [36] found that miRNA-181c was involved in the lipopolysaccharide role of the macrophage inflammatory reaction by regulating Akt1. Importantly, miR-340 was the most significantly altered. Furthermore, *in vitro* study revealed that myocardial cell structure and volume were significantly altered by miR-340 overexpression. It remains unclear whether miR-340 is a ‘cause’ or ‘effect’ of the progression of human heart failure; although, it may help to identify key genes in the global signalling networks that are potential targets for miRNAs and play regulatory roles in modulating molecular networks. Key genes occupy important positions in the signalling networks, and alterations in the expression of these genes by combinations of miRNAs may account for the observed global changes associated with cardiac dysfunction and failure. Cardiomyocyte hypertrophy is a critical pathophysiological change in the course of heart failure. The gene expression levels of ANP, BNP and caspase-3[38] are important indicators for assessing cardiomyocyte hypertrophy. Overexpression of miR-340 significantly elevated the expression levels of ANP, BNP and caspase-3 (*P* < 0.05; Fig. 11). Moreover, miR-340 overexpressed cells showed increased cross-sectional area (*P* < 0.05) compared to the control group. Together, these data suggested that miR-340 overexpression is sufficient to induce cardiomyocyte hypertrophy and change cell structure. The main target gene regulated by miRNAs in this study was IGF1R, which is widely involved in many important biological pathways such as immune response, amino acid phosphorylation and anti-apoptotic effects. After IGF1R binding to its ligand IGF1, two signal transduction pathways are activated: phosphatidylinositol-3 kinase (PI3K)/Akt and MAPK [39], and play important roles in tumour cell proliferation, differentiation and inhibition of apoptosis [40–42].

Taken together, the results of this study demonstrated that five miRNAs were significantly altered in DCM hearts compared with non-failing control hearts. One of the five miRNAs, miR-340, had not previously been identified in end-stage heart failure. Studies on miR-340 overexpressed myocardial cells indicated that it may play a pivotal role in the alteration of global signalling networks during cardiac pathogenesis. This in-depth analysis provides a better understanding of the global regulation of signalling network pathways by miRNAs. This study lays a foundation for the concept that therapeutic interventions targeted at miRNAs may have global effects on signalling networks in heart failure and other disease conditions.
